# Economic value of illegal wildlife trade entering the USA

**DOI:** 10.1371/journal.pone.0258523

**Published:** 2021-10-12

**Authors:** Jia Hao Tow, William S. Symes, Luis Roman Carrasco

**Affiliations:** Department of Biological Sciences, National University of Singapore, Singapore, Republic of Singapore; University of Bucharest, ROMANIA

## Abstract

Illegal wildlife trade is one of the greatest threats to biodiversity. Understanding its economic value is a first step to establishing the magnitude of the problem. We develop a dataset of illegal wildlife trade prices and combine it with seizure data to estimate the economic value of illegal wildlife trade entering the USA. Using 2013 as a reference year, the results reveal that the economic value of illegal wildlife trade entering the USA was, using a conservative scenario where potential outliers were excluded, US$3.2 billion/year (uncertainty range (UR) 5th and 95th percentile of US$0.6–8.2 billion/year) and, without excluding potential outliers, US$4.3 billion/year (UR of US$1.3–9.6 billion/year). Our results for the USA alone are of a comparable magnitude to the lower bound of commonly used global estimates of the economic value of IWT of uncertain origin, suggesting that the global economic value of IWT is currently underestimated and requires an urgent revision.

## Introduction

Overexploitation, unsustainable harvesting of wildlife from the wild, currently stands as one of the main drivers of extinction of global biodiversity [1]. Plant and animal products are traded annually, legally and illegally, to meet consumer demands worldwide [2]. The annual global legal trade of wildlife has been conservatively estimated to value 119 billion USD of the year 2020 [3]. IWT is the world’s fourth-largest illegitimate transnational activity, falling closely behind narcotics, human trafficking, and counterfeit products [4]. In contrast to the legal wildlife trade, due to its illicit nature, the magnitude and value of illegal wildlife trade (IWT) remain poorly understood. Some estimates commonly used are US$7–23 billion/year [5,6], US$5–20 billion/year [7] and US$10–20 billion/year [8]. None of these estimates are however based on open methods and sources in a way that they are reproducible [9].

Here we adopt as definition of IWT the “trade in wildlife or wildlife parts that violates either international legal frameworks or the legislation of one or several of the countries through which a wildlife product has passed” [10]. We define wildlife broadly, including taxa present in the United States Fish and Wildlife Services Law Enforcement Management Information System (LEMIS) dataset, which includes information mostly on vertebrates, invertebrates and, to a lesser extent, plants and microorganisms [11,12].

IWT has socio-economic and health implications that go beyond biodiversity. On the one hand, IWT can undermine the livelihoods of local communities. For instance, iconic species targeted by organized crime syndicates such as elephants and rhinos are critical in sustaining livelihoods based on tourism in rural communities [13]. On the other hand, poor communities may rely on wildlife harvest and trade for their subsistence. For instance, hunting is a key subsistence activity in Hkakaborazi National Park in Myanmar [14] and wildlife trade is a key livelihood activity in rural areas in Madagascar [15]. Wildlife trade can thus range within a broad spectrum of legal to illegal and from sustainable to unsustainable. IWT also increases risks to human health as large volumes of live animals are transported transnationally and, during the process, have contact with trappers, hunters, middle marketers and consumers [16]. This is concerning since most human emerging infectious diseases are caused by virus that have a wildlife origin such as HIV, SARS, and Ebola [17].

To ensure that international trade of wild animals and plants is sustainable and does not jeopardize the survival of species in the wild, the Convention of International Trade in Endangered Species of Wild Fauna and Flora (CITES) entered into force in 1975 [18]. The number of signatories of CITES increased through time and CITES has currently 183 Parties (182 countries and the European Union). CITES provides different degrees of protection to about 35,000 species of plants and animals [18].

Whereas analyses of legal wildlife trade are made possible through the use of CITES data in which CITES Parties report species, products and quantities traded [e.g. 2,19], analyses trying to study IWT are more complex, needing to rely mostly on seizure data. Datasets, where IWT are stored include WorldWISE, managed by the United Nations Office on Drugs and Crime (UNODC) and that stores the illegal trade data collected in the Parties’ annual illegal trade reports [20], specialized datasets like The Elephant Trade Information System (ETIS) that focus on the illegal trade of elephant ivory and other products [21], the new TRAFFIC Wildlife Trade Portal seizure database [22], the European Union Trade in Wildlife Information Exchange (EU‐TWIX) database [23] and the records collated by LEMIS, that track and record IWT seizures entering the USA [11,12].

Past attempts to monitor and quantify IWT using these datasets have increased our understanding of the complexity of IWT. For instance, the patterns in exporters and wildlife products seized entering the USA have been studied using LEMIS [24–27]. A comprehensive account of IWT was made using 12 years of seizure records collated by TRAFFIC, identifying significant trade routes [28]. IWT has also been studied through models attempting to account for seizure underreporting [29]. TRAFFIC seizure records have also been combined with biodiversity transect surveys to understand declines in ploughshare tortoises in Madagascar [30], and the relationships between legal and illegal trade have been studied using a combination of CITES, LEMIS, and EU-TWIX data [31].

Despite the progress in analyzing trade volumes, patterns and species involved, the actual magnitude and economic value of IWT remains highly uncertain [9]. The estimates currently reported in the literature with IWT value per year are varied and either obtained from unreliable sources or derived using unknown methodologies [9]. This leaves a critical gap in our understanding of IWT that hinders our capacity to assess the magnitude of the problem.

To contribute to fill the abovementioned gaps in knowledge on the economic value of IWT, here we aim to estimate the economic value of IWT entering the United States of America.

## Methods

### Data collection

To derive the IWT price dataset, we acquired all seizure records imported into the USA in the year 2013 (year most recently available) from the LEMIS [11] database (http://wildlifetradetracker.org/?db=lemis). An online tool for data download was available at the time the research was conducted. The data are currently available as part of an R package [12]. The data were coded into five categories: species (common name), scientific name, type product, quantity and exporter. Countries with zero quantities of seizures entering into the USA were excluded. We grouped the dataset into unique species-type product combinations (S1 Table). The IUCN Red List category of each species seized were obtained [32]. Species without a threat status were catalogued as NA (this do not include the category "Data Deficient").

Using the species scientific name and type product from the LEMIS dataset in 2013, we searched for prices of each species-type product combination online from September 2019 to April 2020. The search was carried out on the Google Chrome platform using incognito mode, which does not save browsing history, cookies and site data. Fresh caches were used per search on a new incognito page to ensure the search process was not linked to any account profiles or cookies. Each product was searched with the specific standardized format: ‘common name’ OR ‘scientific name’ AND ‘type product’ AND ‘prices’ OR ‘worth’ OR ‘value’ OR ‘for sale’. From the result page, each site was searched in order of appearance until the first price was found, and then recorded. If the site contained multiple prices, due to logistical constraints, up to seven prices were recorded to formulate a range of prices for the product. Then the search stopped. If the site contained a price range, it was recorded and the search would also stop. If the site had only one price, we moved on to the following site and collected more price data following the order in which sites appeared in the results page. We kept checking sites in order until the next price was found, until a total of seven prices or a price range were obtained or until there were no more page entries that contained the search terms.

This process was repeated for all unique species-type product combinations. All the instances of prices per type product and species combination were recorded to later construct uncertainty distributions of the price. The unit of measurement was also recorded (e.g. unit, weight, length), as well as the currency in which the product was sold and year of sale.

The sources used for price data collection involved: prices of IWT of species-specific type products based on published research; prices of legally traded wildlife products found on e-commerce trading sites and prices of IWT of species-type products that were found through news sources.

All prices were corrected for currency conversion and inflation, using an inflation calculator and Bloomberg currency exchange tool [33,34]. All values were expressed as USD of the year 2020.

### Proxies for type products and prices

Several entries did not specify the type of product, and hence we used proxies in a new search to estimate the prices. Some categorizations applied for the assumptions were adapted from past research [35] (S2 Table shows the correspondence between type products and their proxies).

In cases where the prices of specific species-type product combinations could not be found, three types of proxy prices were used instead: replicate proxy prices, substitute proxy prices and average proxy prices. Replica or replicate prices were used for type products that were not available in the market. These were most commonly seen in products such as ‘ears’, ‘skulls’ and ‘trophies’, which were available in the form of reproduction taxidermy. Some examples of such replicates would be life-size reproductions of Loxodonta (African elephant) and replicate skulls of Delphinidae (dolphin) species (all the price proxies used for prices and their type are described in S1 Table).

There were two types of substitute proxies: for species and for type product. For species, price replacements were from the same type products but made from species of the same order, family or genus. For type products price replacement of a specific species, we used other products from that species (S1 Table).

Average proxy price substitution was then performed for two main cases: if the species information was missing (“N.A.” in the dataset), or if the type product of a particular species was listed as ‘unspecified item’. For the first case, the average of the unique type product across the different species in the dataset were used. For the second case, the average prices of the type product in the dataset across species was used.

### IWT economic value calculation

The economic value of IWT for each species-type product combination *i* (*V*_*i*_) was estimated using:

$$V_{i}={price}_{i}\times {unit\;factor}_{i}\times quantity,$$ (1)

where *unit factor* is a factor for each product that converts all units of measurement to a standardized count unit by accounting for the typical dimensions of the items seized. This was necessary as the prices of several products were measured in terms of weight or length, such as meats and skins, while the LEMIS database only displayed whole counts of items seized (*quantity*) for each product. Sources of this factor which constituted one unit were obtained online from the sources used to obtain the prices, using the dimensions of the products sold to represent one unit. Unit factors were categorized as a minimum and maximum factor for each product to reflect the uncertainty involved. Unit factors were multiplied by the price to obtain a price per item [e.g. for type product elephant skin, entry in row 15 in S1 Table, the average price of 45 would be in $/square feet^2^, the unit factor ranges from 10 to 20 (Min_Unit and Max_Unit variables in S1 Table) and would be in square feet^2^/item. Their multiplication would lead to $/item that was then multiplied by the number of items, 12 in this case, to yield a range of values for the seizure of $5,400–10,800]. Product prices that were measured in units or whole corresponded to minimum and maximum unit factor of 1 (S1 Table).

*V*_*i*_ was then divided by inspection rates to account for unobserved IWT that is not seized, leading to a ‘underreporting-corrected *V*_*i*_’. We use the rate at which containers and/or cargo are inspected for IWT at the border, given by the percentage of inspections carried out by patrol officers. This factor was determined using past research on rhino horn and ivory trafficking, which concluded that the inspection rates of containers were less than 5% [36]. Similarly, the detection rates of illegal drug mules at the USA borders were also determined to be no more than 5% [37]. We assumed that conditional on inspection smuggled IWT would be detected and seized. These assumptions are motivated by a lack of data. Ideally, we would have used different detection rates for different taxa.

Summing across all species-type product underreporting-corrected *V*_*i*_ we obtained the economic value of IWT entering the USA in 2013.

### Uncertainty analysis

We attempted to capture the uncertainty in the price dataset and its generation through uncertainly analysis using Monte Carlo simulation and through several uncertainty scenarios.

Using the set of prices obtained for each species-type product combination, we constructed uncertainty distributions of prices to which we applied Monte Carlo simulations. Normal and uniform distributions were used in two sets of simulations. Minimum and maximum unit factors were used to construct uncertainty distributions which were also sampled in every iteration. When negative values of prices were generated by the normal distribution, they were truncated to zero. All uncertainty analyses were performed using the R environment [38]. The sampled price values were then inserted into the steps for species-type product combination value calculation abovementioned. We ran 100,000 simulations for each model (normal and uniform distributions).

As one of the major sources of uncertainty in our analysis was the quality of the price dataset, we further run different uncertainty scenarios to quantify the influence of price source, including potential outliers, and proxy prices on the results. We expected prices from news to be higher as they may be more likely to be reported in the news if they are unusually high. Our price proxies, to populate prices that were not available, could have also influenced the results (see Results for a breakdown of the percentage of prices that used proxies and under which approach). As such, we repeated our economic value estimation under two new uncertainty scenarios: (i) “no news and no outliers” excluding the prices that came from news sources and potential outliers and (ii) “no proxies” excluding the prices that came from proxy prices.

For the identification of potential outliers in the first scenario we used the following criteria:

Identify among the highest prices for a given type product–species combination those that were above the 90th percentile of all highest prices as potential outliers. Among those identified type product–species combination with highest prices above the 90^th^ percentile, remove as outliers in the “no news and no outliers scenario” those that were only supported by less than two observations or those for which the highest price value was more than 1.5 times the interquartile range plus the 75th percentile value of the other price entries for a given type product–species combination.

Our rationale for these outlier exclusions were based on uncertainty (few estimates supporting a very high price) and unusually high price entries compared to the rest of price entries for a given product–species combination.

## Results

According to the LEMIS database, a total of 3,912 seizure records occurred in the year 2013. These seizures corresponded to 2,696 unique shipments. The seizures consisted of 62 unique type products. These type products originated from a total of 566 species, including over nine different taxa (mammals, herptiles, birds, cnidarians, mollusks, fish, annelids, insects, echinoderms). We grouped this dataset into a total of 987 species-type products combinations (S1 Table shows the grouped combinations and S3 Table the seizures broken down by exporting country).

The prices we collected for these species-type products combinations were obtained from news (69, 7%), online trading websites (800, 81%) and research articles (73, 7%). Prices could be found for 84.5% of the species-type products combinations. The remaining 15.5% (153 species-type product combinations) could not be directly found and estimated using proxy prices. Of these proxy prices, 45 (29.4%) species-type products were estimated using average proxies, 76 (49.7%) were estimated using substitute proxies, and 32 (20.9%) were obtained using replicate proxies.

**Fig 1:**
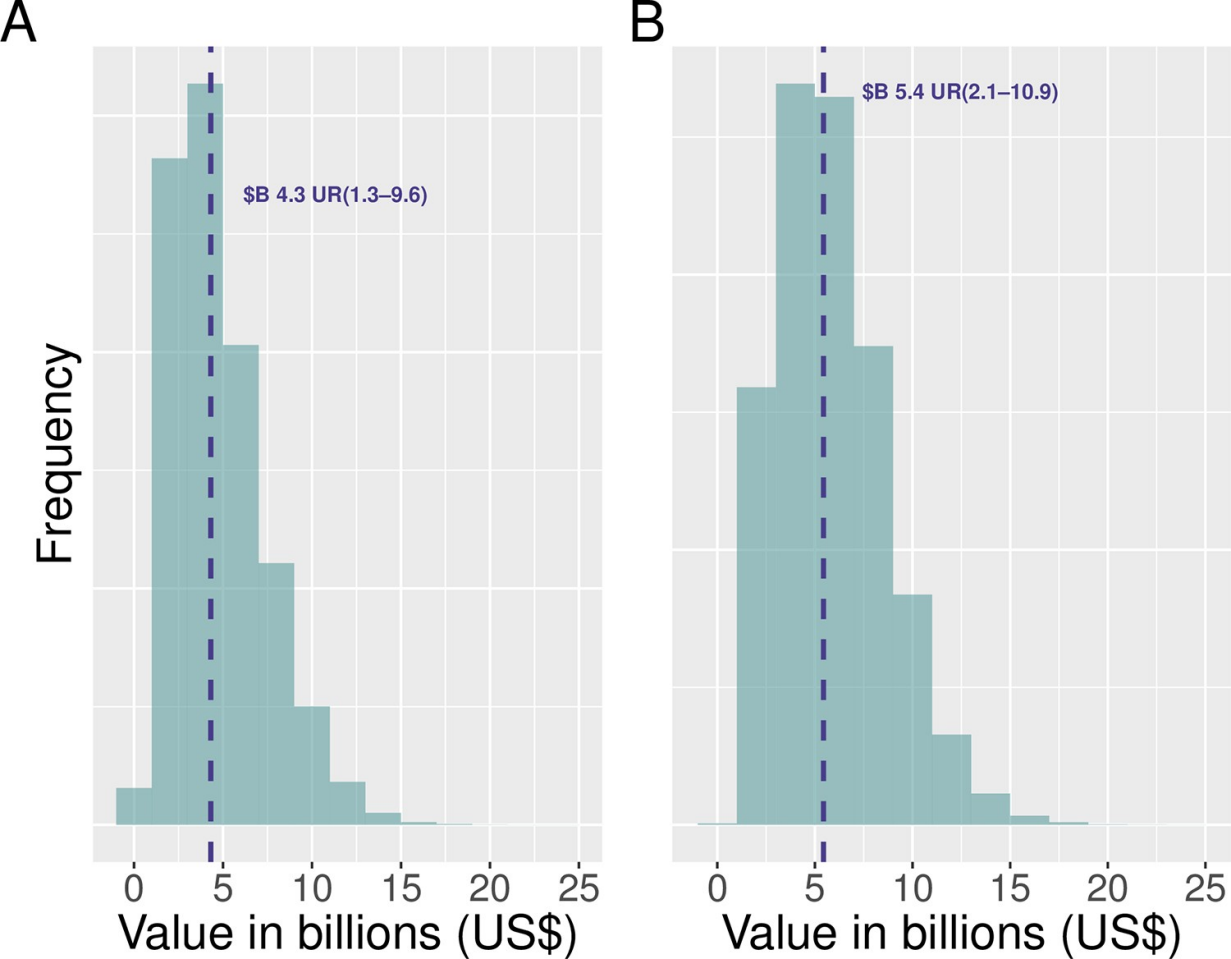
Uncertainty distribution of USA IWT values. A and B: USA IWT economic values using normal and uniform uncertainty distributions respectively.

Using 2013 seizures as reference year, we estimated that the annual mean economic value of IWT entering the USA using normal and uniform uncertainty distributions was US$4.3 billion (uncertainty range (UR) 5th and 95th percentile of US$1.3–9.6 billion) and US$5.4 billion (UR: US$2.1–10.9 billion) respectively (Fig 1A and 1B).

**Fig 2:**
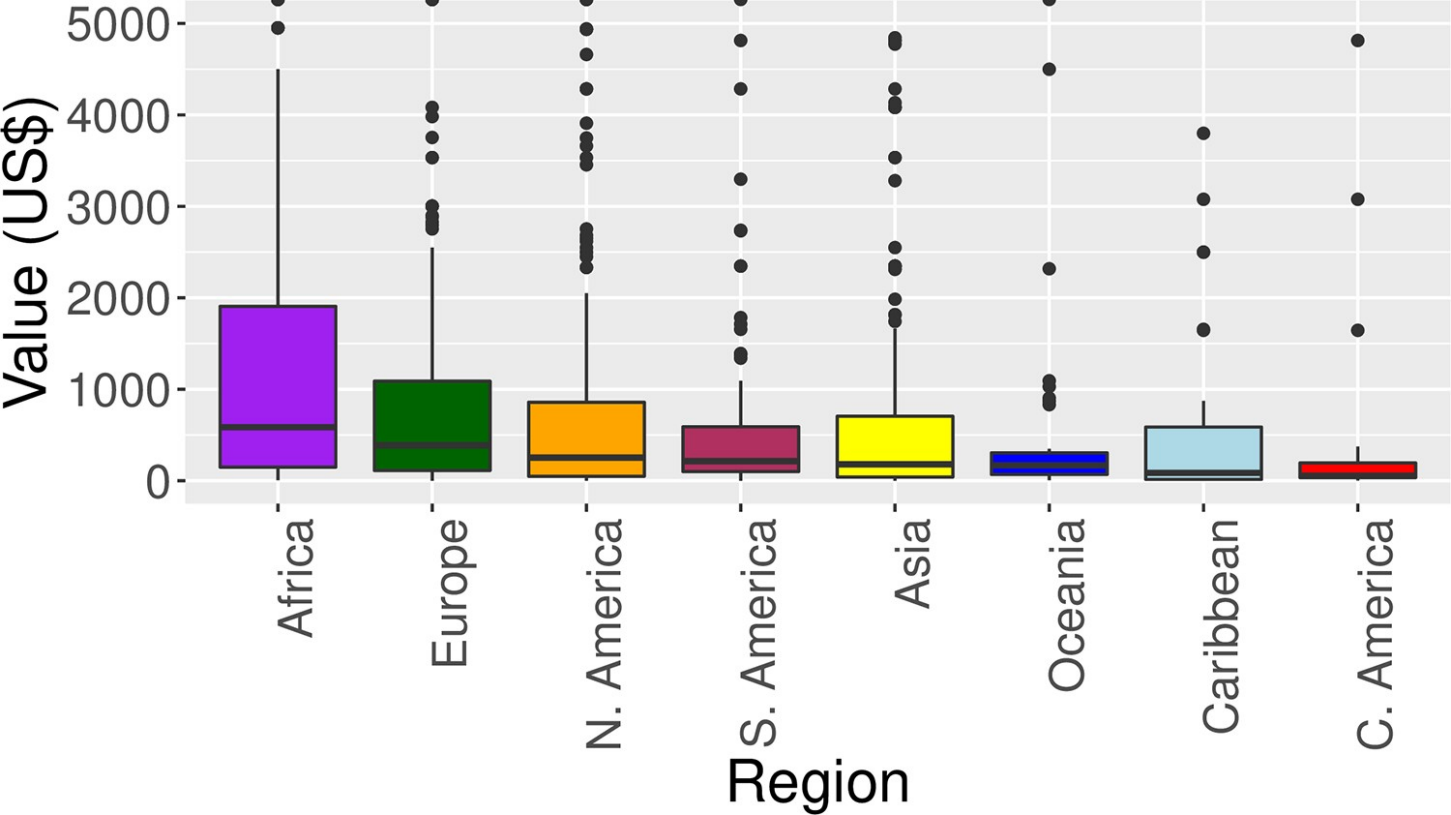
Boxplots depicting average price of seizures into the USA from each region. Values beyond US$ 5000 are not shown.

The region with the highest median seizure price value was Africa (median: 585, interquartile range (IQR): 149–1906, Fig 2). The region with the lowest value was Central America (median: 55.0, IQR: 35–195). The data contained numerous outliers, due to the presence of products with high value such *Panthera tigris* (tiger) trophy and *Panthera pardus* (leopard) skins (Fig 2).

**Fig 3:**
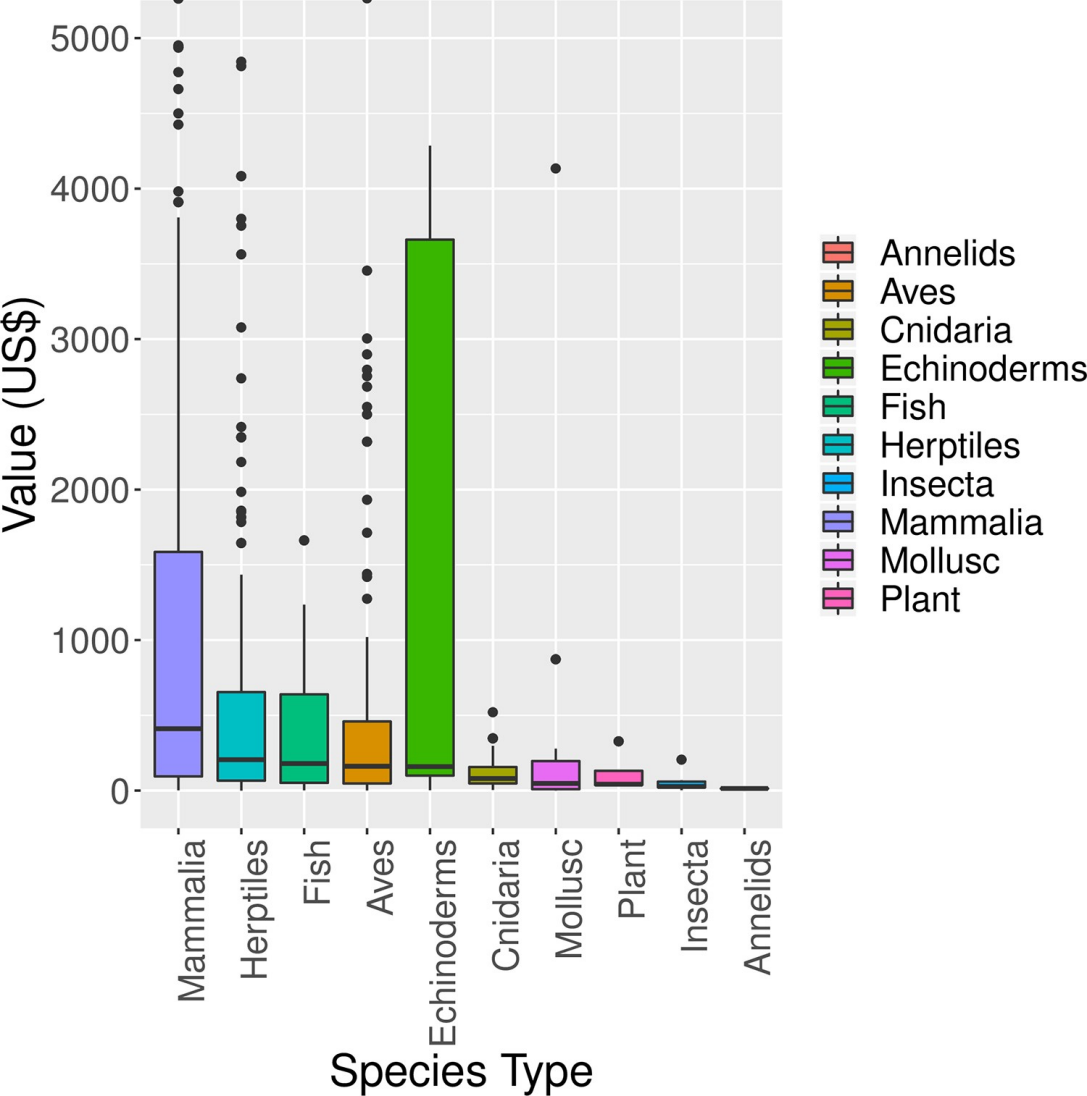
Boxplots depicting average price of seizures into the USA by taxa. Values beyond US$ 5000 are not shown.

Mammal seizures had a higher value (median: 411.1, IQR: 93.7–1585.8) than other taxa except Echinoderms (Fig 3). This value was followed by herptile, fish and avian seizures (Fig 3).

**Fig 4:**
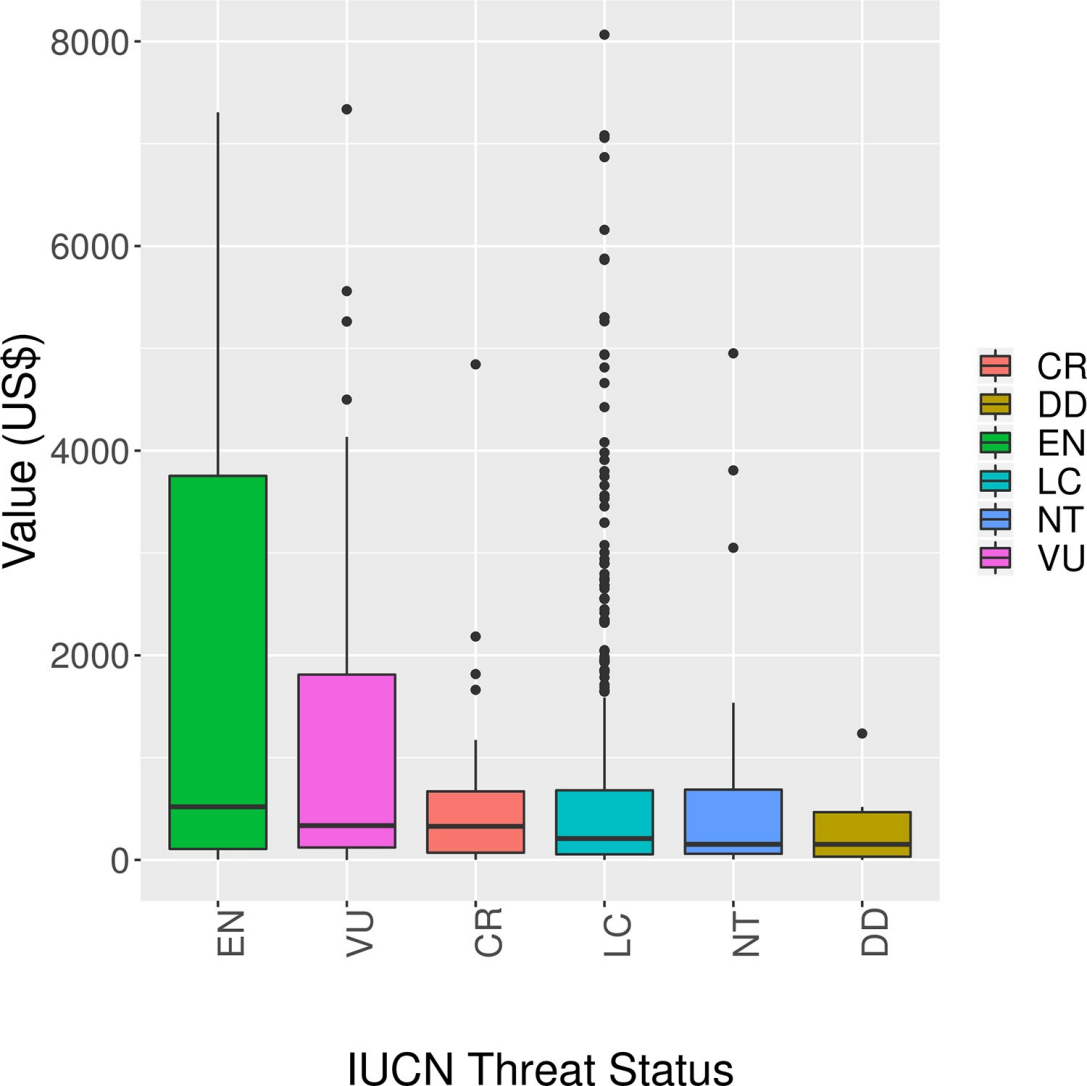
Boxplots depicting average price of seizures into the USA by IUCN threat status. Values beyond US$ 8000 are not shown. DD: Data Deficient, LC: Least Concern, NT: Near Threatened, VU: Vulnerable, EN: Endangered, CR: Critically Endangered.

There were large outliers that represented products of much higher value, such as full-body trophy of mammals or birds, or live herptile specimens (S1 Table). Endangered species products had the highest average value (median: 518.7, IQR: 107–3754) (Fig 4). Near-threatened species products had one of the lowest average value (median: 153, IQR: 60–687) with a maximum value of 4952 (*Equus burchellii* (Common zebra) trophy).

Our uncertainty scenario excluding prices from news and potential outliers led to noticeably lower USA estimates of economic value [US$3.2 billion (UR: US$0.6–8.2 billion) using normal distributions and US$3.4 billion (UR: US$0.8–8.5 billion) using uniform distributions, S1 Fig]. The scenario excluding all type of price proxies led to a small drop in median economic values with respect to the baseline scenario [US$4.1 billion (UR: US$1.1–9.5 billion) using normal distributions and US$5.2 billion (UR: US$1.9–10.7 billion) using uniform distributions, S2 Fig].

## Discussion

We estimate that the median economic value of IWT entering the USA in 2013 to be US$4.3 billion (uncertainty range of US$1.3–9.6 billion using normal uncertainty distributions). Our median results of economic value for the USA alone are very close to the lower range, and of the same order of magnitude, of global estimates, with our upper range of uncertainty within some of the most commonly cited range of economic values for global IWT of US$7–23 billion/year [5], of unknown origin and/or methodologies [9]. Our results thus suggest that previous global estimates may be underestimating the global value of IWT, even after considering that previous studies have shown the major role of the USA in the IWT market [39]. Our estimate of economic value of IWT entering the USA is further likely to be an underestimate if we consider the trend in increasing number of global seizures. For instance, the number of global seizures of 2013 (12,751), were substantially higher in 2017 (latest available value of 20,762) [20]. Assuming that this increase in seizures occurred due to increased trade volumes and not improvements in reporting standards, the economic value could almost double. This increasing trend is however likely explained as well by the new CITES illegal trade reporting requirement into WorldWISE introduced in 2017 [20]. This large uncertainty calls for further research to update the economic value of IWT entering the USA as data on more recent years become available. Further research could also consider our methodology to expand analyses to species-type product combinations beyond the USA towards a global estimate. A global estimate would require data on IWT across all possible country origin–destination pairs to capture different IWT dynamics. For instance, the dynamics in SE Asia would be expected to be quite different to wildlife trade entering the USA as the major importers of legal wildlife trade from SE Asia are the EU and Japan [40].

It is difficult to put our estimates of economic value of IWT entering the USA into perspective. On the one hand, assuming that IWT can play a role in the emergence of zoonotic infectious diseases of pandemic potential, our estimates of the economic value of IWT entering the USA would be dwarfed by the large potential damage, suggesting that investing in the control of IWT—currently conservatively estimated to be at least USD185 million per year [4]—could meet with high returns on investment—a suggestions that chimes in with recent analyses [41]. On the other hand, a fraction of IWT plays a critical role for food security and livelihoods in poor communities that are more reliant on natural resources extraction [e.g. 14,15]. In addition, IWT may not be necessarily unsustainable, being essential to have a nuanced approach in the identification of the actors involved, their values and the distribution of benefits from IWT. Traditional approaches to reduce IWT have focused on increasing law enforcement at the state level [42,43]. Yet, community engagement and investment is increasingly recognized as crucial to not only intervening at the ground level of poaching and trafficking, but also in shifting perceptions of local people toward supporting conservation rather than unsustainable IWT [44].

The average prices of IWT seizures by taxa revealed the higher average economic value of mammalian seizures in the USA. This was also partly due to the larger proportion of trophy and taxidermy products seen in mammalian seizures. Trophies and taxidermy products made from large mammals are commonly more expensive due to the value attached to them [45]. Size and shape of a trophy are major factors of its value, which would justify why common mammalian trophies such as bovids would have a higher value, as they are comparatively larger than species of other taxa [46]. Another reason for the higher average economic value of IWT was due to the presence of high-value mammalian IWT products such as ivory, bones and skins of big cats, and bear bile [3]. In contrast, avian seizures consisted primarily of feathers of different species, which are not as lucrative as these mammalian seizures. High prices are also possible in other taxa, for instance the totoaba in the Gulf of Mexico for which high demand for its swim bladder in the Asian market—up to $10,000 per bladder—drives its price very high. The high fishing pressure on the totoaba is also driving the rarest marine mammal, the vaquita, to extinction through the indiscriminate use of gill nets [47].

It should however be noticed that prices from news sources also had a role in some unrealistic prices for iconic species. For instance, we recorded a price of 234,000 for *Panthera pardus* (leopard) skins with a price of $819,000 for *Panthera tigris* (tiger) trophy, which are very likely inflated and were excluded under an uncertainty scenario that did not considered estimates from news sources and potential outliers. This perception is corroborated by the drop in IWT global economic value once news sources are excluded. In contrast, our economic value estimates of IWT entering the USA remained in the same order of magnitude after proxy prices were excluded, still suggesting the value of IWT is likely severely underestimated. All things considered, the quality of the price data remains the area of greatest potential for improvement in our analyses and should be a matter of future research.

A key limitation is that we used only online price data. These online data partially capture actual market prices from research reports but the major contributors to price were online sales. This is expected as data on real IWT transactions are very scarce. Using online sales further implies conflating legal and illegal prices, which may be quite different as illegal markets would be expected to have a different equilibria than legal markets [48]. In addition, we could not distinguish the level of the supply chain each price corresponded to. Online sales are likely to reflect final consumer paid prices and not prices received by poachers, trappers and intermediaries. Relatedly, our search terms were in English, probably biasing our estimates towards international markets and final consumers. Future work collecting these data from physical markets and online platforms [e.g. social media using local languages [49]] is likely to be costly but necessary to further shed light on the distribution of benefits of IWT among the different actors involved. Paucity of price data for many species–type products combinations meant that the uncertainty distribution could not be meaningfully estimated from data. Our approach was to compare the results using normal and uniform distributions, which led to relatively similar results.

Our approach of estimating value was done by multiplying prices times quantities of IWT. This approach is a simplification as, ideally, we would have knowledge of the supply and demand curves of each species-type product combination to estimate the consumers and producers’ surplus involved in the market of each IWT product. In reality, the supply and demand curves of illegal products are unknown, leaving our simpler approach as the only viable option. Our approach implies a vertical supply curve and an horizontal demand curve and is commonly used in studies involving a large range of goods and services for which markets are not known [e.g. 50,51]. Future work directed towards characterizing the supply and demand of specific legal and illegal wildlife products would be useful to forecast how the market may react when different interventions may affect the price of a product [48]. This is essential given the clear links between increases in prices and surges in supply of legal and illegal wildlife trade as observed, for instance, in South Africa [52].

Our analyses present other limitations. The LEMIS dataset consists only of detected and reported IWT. In reality, large volumes of IWT go undetected in what is termed the ‘dark number’ of IWT [3]. This large proportion of undetected IWT could have its roots in IWT not being the main priority by customs inspectors as shown in the case of Norway whereby wildlife seizures are often accidental [53]. The implicit assumption in our analyses is that non-detection is distributed at random across species–type products combinations [25]. This assumption is unlikely to be true and would require further research. For instance, it would be expected that higher emphasis on detection is placed on CITED-listed animals as opposed to CITES-listed and non-listed plants which get substantially less attention [54], making us underestimate the value of the trade corresponding to plants. The proportion of the seizures corresponding to live animals versus animal parts, which would play a part in the probability of detection, also varies widely by taxa, with live birds and reptiles seizures being common while the proportion of live seizures for mammals is low [55]. Incomplete records in live seizures with 70% of countries party to CITES failing to provide data to CITES has also been raised [56]. The mode of transport (e.g. via cargo vs. personal baggage) is also likely to have associations with different taxa and influence probability of detection [26]. LEMIS reports the country where the seizure was exported from but not the actual country of origin [25]. For instance, exports from Europe into the USA in tropical species need to be regarded in light of this limitation as we would expect that European countries are acting as transit points and not actual origins of the trade. Indeed, EU countries have been identified as both an important destination and transit for IWT [55]. Many seizures are not identified at the species level or lack information on type of products. We tried to overcome these data gaps with proxies. Our uncertainty analysis shows that our results, at the aggregate level, were not severely affected by these proxies. In addition, there are unavoidable time mismatches in the data that are worth noting. We generate the list of species-type products combinations from LEMIS data in 2013 while generated the prices dataset from the year 2020. Our results could thus be different to the extent that species-type products and their quantities involved per seizure changed from 2013 to 2020. Further research could also attempt to expand the identification of species–type products combinations using years different from 2013 as new data become available.

## Conclusions

We estimate that the economic value of IWT entering the USA in 2013 was US$4.3 billion (uncertainty range of US$1.3–9.6 billion). Our estimates for the USA alone are in the same order of magnitude of previous global estimates of unknown methods and origin, suggesting previous global studies underestimate the economic value of IWT. Our results also show that endangered mammal species from Africa present higher prices—sometimes several orders of magnitude higher—than other taxa and threat status. Our approach offers, to our knowledge, the first publicly available estimation of the economic value of IWT entering the USA and paves the way for future work to estimate global values of IWT. Yet the limitations of our work are many and we hope our study can serve as motivation to more refined estimates of the economic value of IWT.

## Supporting information

S1 Fig Uncertainty distribution of USA IWT values after excluding prices from news sources. C and D: USA IWT economic values using normal and uniform uncertainty distributions respectively.
(PDF)

S2 Fig Uncertainty distribution of USA IWT values after excluding proxy prices. C and D: USA IWT economic values using normal and uniform uncertainty distributions respectively.
(PDF)

S1 Table Type products and species seized in 2013 in the USA. Corresponding prices, unit factors for conversion to unit prices, proxies and substitutes used to input prices.
(CSV)

S2 Table Categorization for product assumptions and closest related item. (DOCX)

S3 Table Break-down of imports of species-type products by country of origin. (XLSX)
