# Supplementary figures and images for: Economic value of illegal wildlife trade entering the USA
Source: PLoS One. 2021 Oct 12;16(10):e0258523. doi: 10.1371/journal.pone.0258523 (PMC8510001; doi:10.1371/journal.pone.0258523)

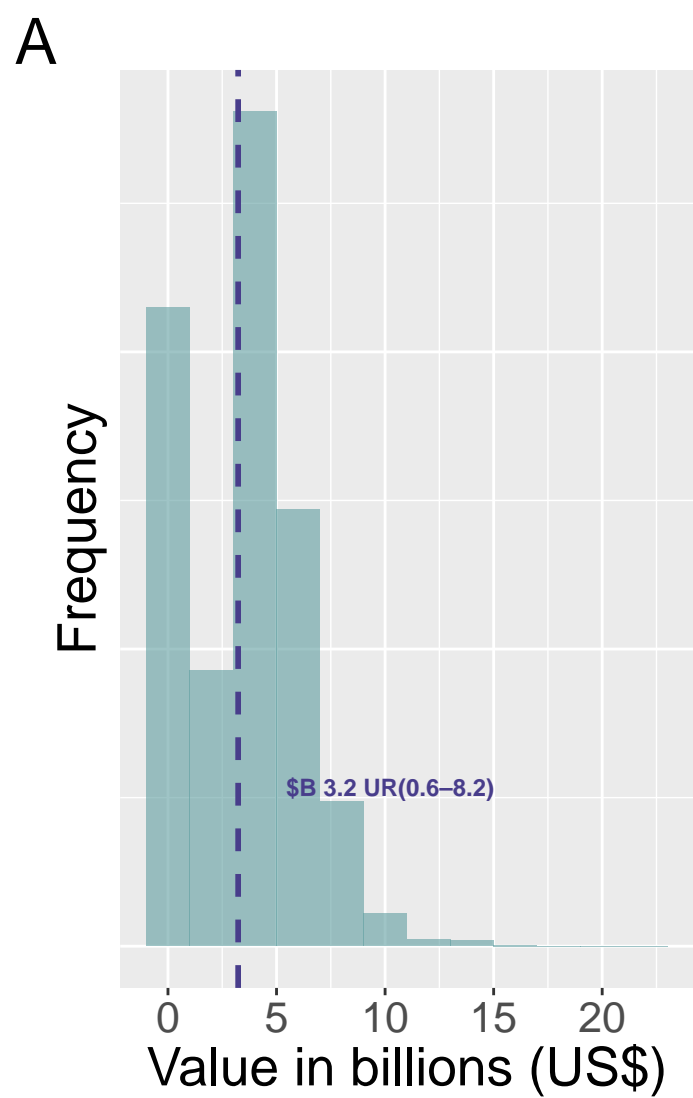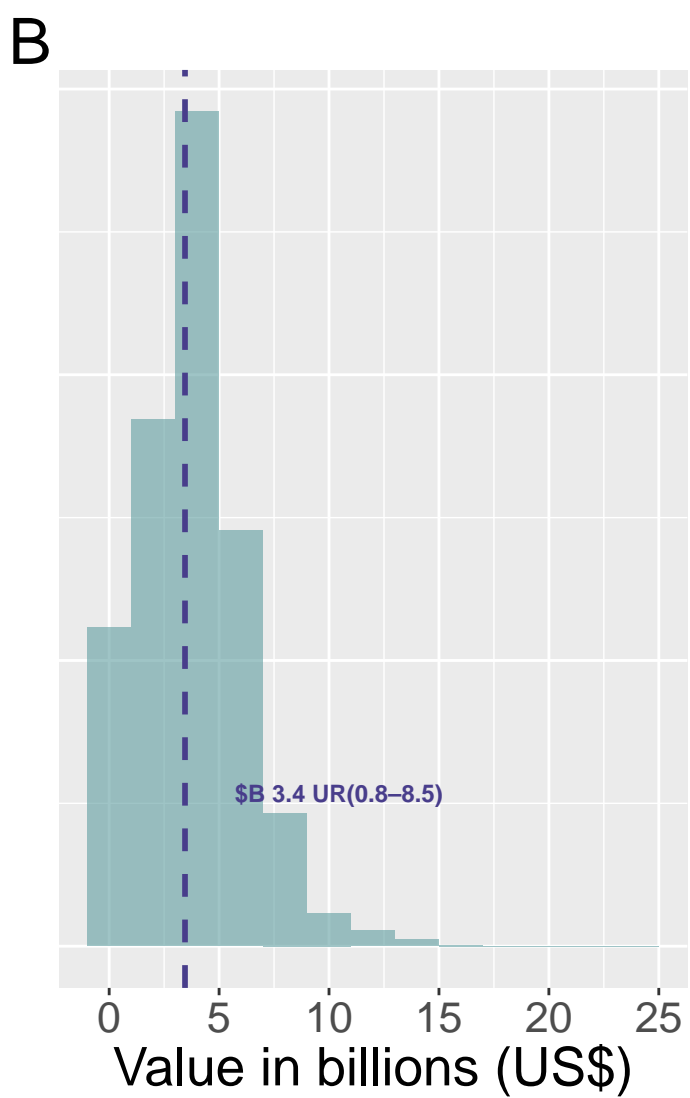

Supplement: S1 Fig — C and D: USA IWT economic values using normal and uniform uncertainty distributions respectively. (PDF) [file pone.0258523.s001.pdf]

A

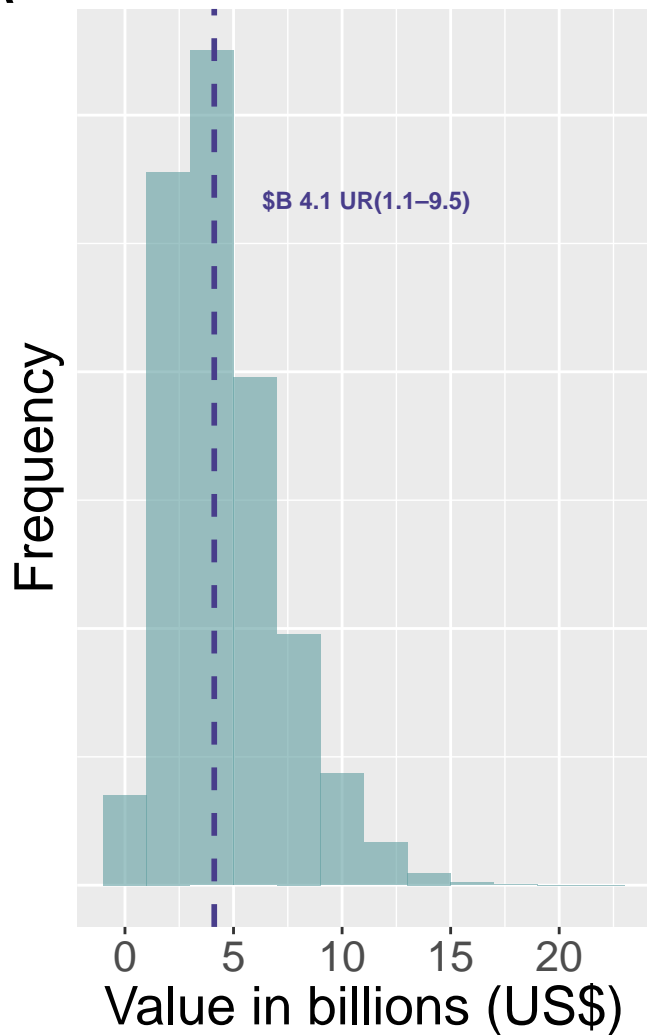

B

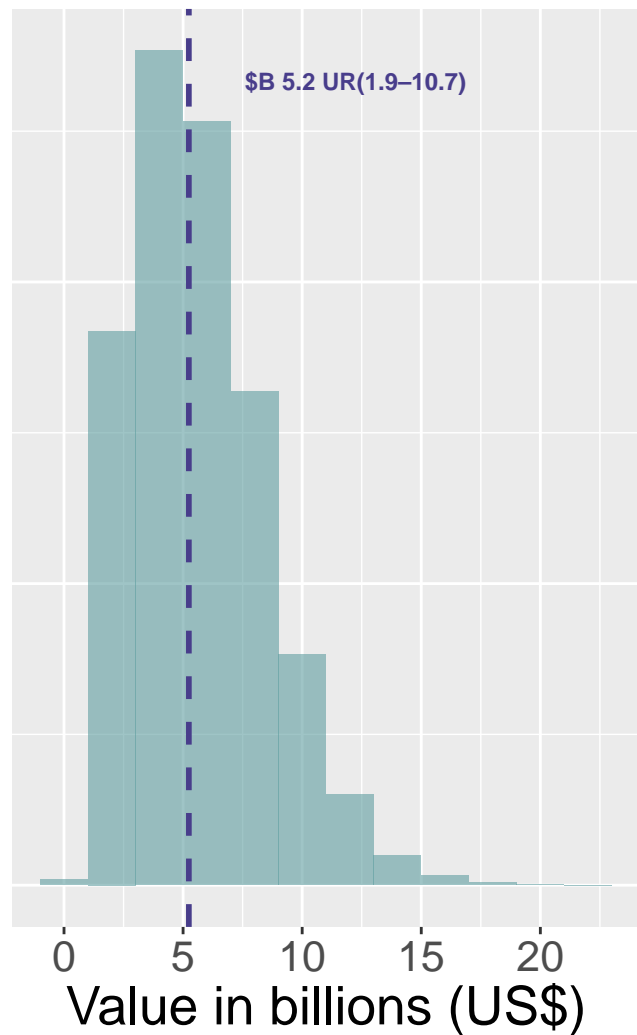

Supplement: S2 Fig — C and D: USA IWT economic values using normal and uniform uncertainty distributions respectively. (PDF) [file pone.0258523.s002.pdf]
