# Supplementary material for: Economic value of illegal wildlife trade entering the USA
Source: PLoS One. 2021 Oct 12;16(10):e0258523. doi: 10.1371/journal.pone.0258523 (PMC8510001; doi:10.1371/journal.pone.0258523)
Supplement: S2 Table — (DOCX) [file pone.0258523.s004.docx]

Table S2. Categorization for product assumptions and closest related item.

| **Type Product** | **Assumption Made** | **Closest related item** |
| --- | --- | --- |
| ‘Small leather product’ | Wallets, belts, money clips, bracelets | - |
| ‘Large leather product’ | Duffel bags, handbags, bags, leather coats | - |
| ‘Jewelleries’ | Any jewellery available (rings, necklaces, bracelets, pendants) | - |
| ‘Trophy’ | Taxidermy products (Shoulder mounts, full-body mounts, half mounts) | Skulls, bones |
| ‘Whole animal (dead)’ | Taxidermy, meat, live specimen (died during transportation) | - |
| ‘Bone’ | Bones, skulls, full skeletons | - |
| ‘Bone carvings’ | ‘Bone’ with carvings on the product/carved products | ‘Bone’ |
| ‘Horn’ | Horn of species | - |
| ‘Horns (whole)’ | Entire pair/set of ‘Horn’ | ‘Horn’ |
| ‘Horn Carving’ | ‘Horn’ with carving/carved products | ‘Horn’ |
| ‘Ivory’, ‘tusk’ | Ivory tusk/tusk of the species, teeth of the species | - |
| ‘Ivory carvings’ | ‘Ivory’/ ‘Tusk’ with carvings/carved products | ‘Ivory’ |
| ‘Shell products’ | Product made from/using shells of species (necklace, sculptures, rings) | ‘Shells’ |
| ‘Ders’ | Derivatives | - |
| ‘Extracts’ (depending on species) | Medicinal products, oils, derivatives of species, body parts of species | - |
| ‘Cavs’, ‘Eggs (dead)’ (species-dependent) | Caviar | Eggs (dead) |
| ‘Clothing’, ‘Garment’ | Garments made from the skin/fur/feather of species | Small/Large leather products |
| ‘Skin pieces’ | Cut pieces of skin/leather of species | ‘Skins’, ‘Trims’ |
